# A new Monte Carlo simulation tool for designing an archaeological landscape sampling strategy

**DOI:** 10.1016/j.mex.2020.101124

**Published:** 2020-11-04

**Authors:** Amy Tabrett, Amy Mosig Way

**Affiliations:** a School of Philosophical Inquiry, Faculty of Arts and Social Sciences, The University of Sydney, Building A14, Quadrangle University, Sydney, NSW 2006, Australia; b The Australian Museum, 1 William Street, Sydney NSW 2010, Australia

**Keywords:** Sampling, Monte Carlo simulation, Site detection, Archaeology

## Abstract

This article describes in detail how the Dig It, Design It (DIDI) simulation tool operates to design a subsurface landscape sampling strategy and predict its likely effectiveness. The purpose of the DIDI model is to help archaeologists develop statistically sound subsurface sampling programs that maximise the number of sites found while minimising the number of sampling units used. It has been unusual for archaeological test-pitting programs to be theoretically tested or statistically justified by simulation prior to implementation. Previous research by Kintigh (1988) and Krakker et al. (1983) established the statistical principles underlying the subsurface sampling of a rectangular survey area, and Kintigh pioneered the use of Monte Carlo simulations to test the effectiveness of these sampling strategies. DIDI provides an updated version of this simulation approach that has three key benefits over the original version:

1. It allows the user to model a larger range of possible archaeological conditions by providing an additional density distribution function (see below), and making the clustering parameter available with all distribution functions;
2. DIDI gives the user the option of filling the previously unavoidable gaps around the edges of the survey area with additional, suitably placed test-units, thereby increasing the detection rate of a sampling strategy; and
3. It is free to download and use and is compatible with modern operating systems.

## Graphical abstract

**Figure:**
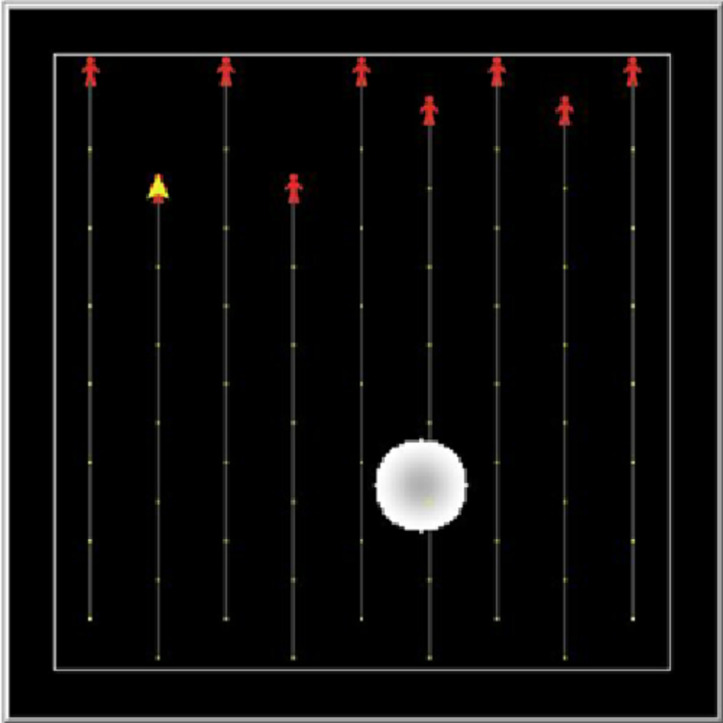

**Table:** Specifications Table

Subject Area:	Earth and Planetary Sciences
More specific subject area:	*Archaeology*
Method name:	*Dig It, Design It* *Way, A. M. and A. Tabrett (2018). "Dig It, Design It: A new simulation tool for the design of optimal subsurface testing programs." Journal of Archaeological Science: Reports****21****: 158–165.* DOI: 10.1016/j.jasrep.2018.07.007
Name and reference of original method:	*Kintigh, K.*[1]*. "The effectiveness of subsurface testing: A simulation approach." American Antiquity****53****(4): 686–707.*
Resource availability:	*DIDI is free to download from*www.DigItTools.net*and needs NetLogo version 6 already installed to run (also free from*https://ccl.northwestern.edu/netlogo/download.shtml*)*

## Method details

The standard archaeological approach to investigate a survey area for subsurface archaeological material when there are no visible remains on the surface is to dig small test pits throughout the area in the hope of intersecting a site. Most archaeologists have not, however, used a statistical approach to determine the best placement of these pits – one that minimizes the number of pits they need to dig while maximizing the chance of intersecting sites. Consequently the effectiveness of the test pitting programs used by archaeologists has rarely been evaluated or scrutinized, and it has been highly unusual in most countries for archaeologists to consider or publicize an estimated detection rate for a proposed test pitting strategy. The general concepts and method for how to estimate a detection rate were developed in the 1980s [1,2] but the software developed by Kintigh has for many years been impossible to use with modern computer operating systems. In response to this problem, Way and Tabrett [3], [4], [5] recently developed two free simulation tools to help archaeologists design an optimal subsurface sampling strategy for their survey area and check the effectiveness (in terms of detection rate) of any such strategy employed by themselves or others. While the statistical and geometric principles governing the creation of an optimally spaced test-pit grid have been discussed elsewhere [1,^2^,4], here we describe in depth the how the simulation tool Dig It, Design It (DIDI) operates, point the reader to additional user resources available, and explain how several key improvements were incorporated into the programming.

DIDI can be downloaded for free from http://www.digittools.net. It was written in NetLogo 6 [6] which is also available for free from https://ccl.northwestern.edu/netlogo/download.shtml. The DIDI model operates by providing the user with the most effective test pit layout for discovering sites with particular characteristics in their survey area, and then repeatedly simulates the use of this layout to detect randomly placed sites. The user can specify the size of these sites, the distribution of artefacts within them, and their average artefact density. The strategy's performance during simulations is used to estimate its overall detection rate (percentage of simulation runs when the layout detected a site) for sites with the characteristics tested.

DIDI contrasts to most existing geostatistical methods (e.g., ArcGIS Geostatistical Analyst tools) in that it requires no initial data to inform the model; the user can simply test a range of chosen scenarios to investigate the effectiveness of different sampling strategies in detecting the presence of a target object (‘site’ in archaeology) in a 2D area. This is a particularly useful feature for fields of study like archaeology where a landscape may need to be sampled for the first time and no relevant data exists describing what is likely to be found. DIDI provides a statistically rigorous starting point for such investigations, as well as for those where more information exists about the presence of the target object in a landscape, and the nature of the object itself. DIDI also differs from geostatistics in that it models the detection of an individual site during each replicative iteration, and therefore is not concerned with the spatial relationship of multiple sites.

Although DIDI is not an agent-based model, the below description follows the ODD reporting protocol outlined by Grimm et al. [7] because its structure is highly suitable to describing how the model operates.

## Scales, entities and state variables

Because DIDI was written in NetLogo, survey areas are conceptualized as ‘worlds’ made up of many smaller, identically sized square units, or ‘patches’. This was appropriate for an archaeological context because archaeologists typically work with a grid when approaching any excavations. The model is hard-coded at a patch scale of 0.25m^2^. This was judged to be a useful minimum test pit size because pits smaller than 0.5 m by 0.5 m are rarely dug in archaeology. The user can also simulate the digging of larger pits (currently 4m^2^ is the largest size the model allows). The size of the survey area to be tested in the model is inputted by the user. Currently in the design phase of a test excavation strategy, only square or rectangular areas can be tested. More work is needed to establish the most effective test pit layouts for survey areas of other shapes. While the size of the survey area which can be tested in DIDI is theoretically unlimited, large areas will make the model run slower so it is recommended that if a large area is to be surveyed, it should be divided into smaller areas for simulation.

The entities used to conduct the simulation in the model are archaeologists (who create the grid of test pits), test pits, and sites. The archaeologists are only present because they provided an easy way to select the patches that are to be test pits; they have no characteristics and die after performing this function. Test pits and sites have the state variables listed in Table 1. While the site characteristics are largely determined by the user, many of the variables relating to the test pits are determined by those of the sites in the same location. This is how the model detects sites. Each time the simulation runs, the time increments by one, providing an easy way to track the progress of the simulations.

**Table 1:** The state variables used to characterize the main model entities.

Entity	State Variable
Pit	intersected (binary variable)
	detected (binary variable)
	sites per pit (to monitor any over-detection)
	mean pit artefact density
	detection probability
Site	r (distance from any patch within the site to the site centre)
	artefact density at r

## Process overview and scheduling

Prior to running a series of simulations, the 2D NetLogo world is resized to match the dimensions of the user's survey area. Then, each simulation run is scheduled in the following way:

1. Any existing pits or sites are cleared, and time is reset to 0.
2. A randomly placed site with user-defined characteristics is generated.
3. Archaeologists are spawned, place test pits of appropriate dimensions (user-defined) within the survey area according to the grid parameters calculated using the input data (see Section 1.4), and then die.
4. Test pits intersecting the site are identified, and their binary ‘intersected’ state is changed. The density of artefacts at the point of intersection is also shared with the test pit. If test pits are larger than one patch, a mean density of artefacts within the pit is calculated. The mean density of artefacts within the test pit is used to calculate whether or not the site is detected by that pit (see Section 3 for the details of these calculations).
5. If at least one test pit has detected the site, the detection counter is increased by one. Regardless of whether a site is found or not, the tick counter increases by 1.
6. Steps 1–5 are repeated until the required number of simulations has been performed.
7. The results of the simulations can then be exported in .csv format, including the grid parameters used and the coordinates of the test pits simulated.

## Design concepts

In order to model the possible variation in the archaeological record of a survey area, it was important to incorporate stochasticity in the generation of sites. Following Kintigh [1] and Krakker et al. [2] the model does this in two ways. First, when sites are generated, a seed patch is chosen randomly by NetLogo to be the centre of the site. Sites are modelled as circular, but in NetLogo the world (and therefore these circles) get divided into a number of square ‘patches’ 0.5 m in length and width. This gives sites a pixelated effect when small, but as they get larger the approximation of a circle improves. This pixilation effect was deemed reasonable for a model of archaeological test pitting because the pit is often the smallest spatial unit used in test excavation of a landscape, and the location of artefacts within the horizontal space of the pit remains unknown. Depending on the settings chosen by the user, the selection of a seed patch can be from within the survey area only, or it can be from somewhere within the survey area or within an additional boundary strip around the edge (equal to the radius of the site). This latter possibility allows the user to test their pit layout against the possibility of sites being only partially contained by a survey area (which is the most realistic scenario). Over large numbers of simulations, each patch in either of these site generating regions has an equal probability of being selected as the centre of a site.

Second, randomness is incorporated at the point where site detection is determined (Step 4 in the model process schedule above). DIDI uses the mean artefact density within a pit in conjunction with either the Poisson distribution (if artefacts are clustered) or the negative binomial distribution (if artefacts are not clustered) to calculate the probability of the site being detected in this pit (details in [4]). If a randomly chosen number between 0 and 1 is less than or equal to this probability, then a test pit detects the site. In general for both distributions, the higher the density of artefacts in a pit (and therefore in a site), the more likely a site is to be detected.

The grid of test units drawn by the archaeologists in the simulation is arranged in a sequence of adjacent equilateral triangles (i.e., a hexagonal grid) because this has been shown to be the most effective for rectangular survey areas [1]. At this point in time the model does not allow any variation to this layout (because the survey area must be square or rectangular), but the effectiveness of this layout for detecting sites in unusually shaped survey areas can be ascertained post hoc using Dig It, Check It [3]. DIDI is programmed to position this hexagonal grid of pits within the survey area, where the pits are spaced just close enough together to intersect a site of the size entered by the user. It arranges the grid so that it is equidistant from all survey area edges, with the full width and length of all pits fitting within the survey area.

**Fig. 1:**
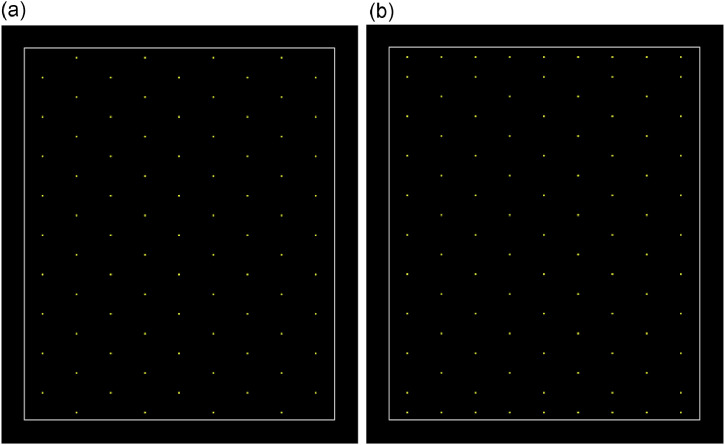
a) A hexagonal grid of test pits with some gaps in the grid at the top and bottom edges; b) the same hexagonal grid with these gaps filled by additional test pits.

A useful new feature offered by DIDI is the filling of gaps in the grid around the perimeter of the survey area. These gaps arise because of the relationship between the survey area dimensions and the site size being modelled. If the survey area dimensions are not multiples of the spacing between pits needed to just intersect a site, then the pit grid needs to be offset from the edges of the survey area. Sometimes this can produce gaps in the grid which can decrease site intersection and detection rates (Fig. 1a). DIDI has a switch feature that when turned on tells the archaeologists to place pits in these gaps aligned with the rest of the grid (Fig. 1b). While this means a greater number of pits must be excavated, it almost guarantees detection of all sites (depending on site densities of course). Whether this improvement is worthwhile must be assessed by the user.

In order to design the optimal grid of pits and estimate its rate of detection for sites of any size, average density, or density distribution function, the user needs to be able to control the parameters relating to site characteristics (hopefully based on pre-existing archaeological evidence or archaeological models). These parameters form part of the initial model setup and are discussed in the next section.

## Initialization and input data

As mentioned elsewhere [1,4], simulation of site detection rates requires input from the user relating to the characteristics of sites that they predict to be present in a survey area. The site characteristics required by the user for the initialization phase of the model are listed in Table 2. The density distributions available in the simulation tool are a mixture of those which are theoretically appropriate (as per [1]), and those that have been found to be useful descriptions of artefact spatial distributions by past work (e.g., Way [8,9]). The availability of this latter type of distribution is unique to DIDI and we hope to make further distributions available in response to future research. This will serve to increase the accuracy of the detection rates predicted and make DIDI a more flexible modelling tool as knowledge of the spatial distribution of artefacts in archaeological sites improves. For similar reasons, the clustering parameter *k* has been made available for use with all density distribution functions.

**Table 2:** The site characteristics required by the model.

Site Characteristic	Description
Diameter	The diameter of the site to be discovered; in metres
Average artefact density	The average density of artefacts likely to be found (in artefacts per square metre)
Density distribution	The 2D function which best describes the distribution of artefacts if a straight line is cut through the centre of the site from one edge to the opposite edge.
Clustering parameter k	The degree of clustering exhibited by the artefacts within the density distribution. The modelling of clustering can be turned off and on by a switch.

**Fig. 2:**
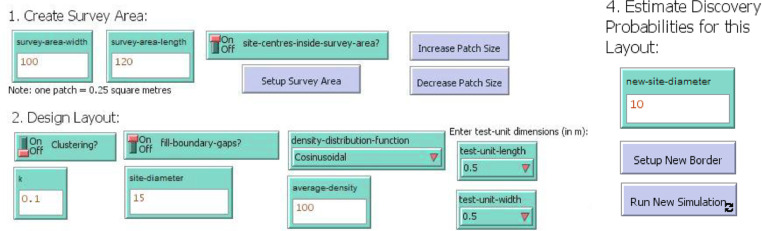
The inputs required as shown in the DIDI interface.

The user is also required to enter the dimensions of the survey area they wish to simulate. Fig. 2 shows the input tools for all model parameters as they appear in the user interface. Steps 1 and 2 are used to design the optimal layout for the survey area, while Step 4 holds this layout constant and changes the size of the site being simulated.

## Output data

DIDI produces several kinds of useful data. Most importantly, at the end of a sequence of simulations DIDI determines the most effective test pit layout for the detection of sites with particular characteristics. These estimates, along with the initialization data and parameters describing the grid itself (i.e. the setup conditions of the simulations) can be exported as a .csv file upon completion of the simulation sequence. The performance of the chosen grid can also be tested against different site characteristics (e.g. sites with a smaller diameter), to determine how effective the designed strategy would be in the detection of other site types which may also be present in the study area. These results can be similarly exported. The user is also able to export the locations of the pits to be dug as a .csv file, and a .jpg map of the layout in the survey area. These output files are designed to make it as simple as possible for the user to apply the digital pit layout in real life, and to justify their choice of subsurface sampling strategy.

## Method validation

Table 3 show how estimated detection rates for a single layout vary with site characteristics. The test unit spacing was kept constant throughout these simulations (it was designed to be optimal for the first site diameter, 10 m), and the survey area investigated was 100 m by 100 m. No artefact clustering was simulated, and square pits of 0.25m^2^ were dug. As expected, detection rate decreases with artefact density and the diameter of sites generated during the simulation. More concentrated density distribution functions (e.g., LGR (Lake George Regression), Cosinusoidal) have lower densities of artefacts around their margins, and therefore pits need to be more closely spaced to achieve both intersection and detection for these. Adding pits in the gaps around the boundary increases the intersection and detection rates.

**Table 3:** Changes in estimated site detection rates in response to changes in site size, density and density distribution function, as calculated by DIDI.

Generated Site Diameter (m)	Average Artefact Density	Boundary gaps filled?	Density Distribution	Number of Simulations	Estimated Intersection Rate (%)	Estimated Detection Rate (%)
10	20	No	Uniform	1000	89.1	88.5
10	20	Yes	Uniform	1000	92.2	92.0
10	20	Yes	LGR	1000	90	39.8
10	5	Yes	LGR	1000	91.5	24.0
5	20	Yes	LGR	1000	33.3	12.5
5	5	Yes	LGR	1000	32.7	5.8

## Additional information

A step-by-step video of how to set up and use DIDI is available at https://www.youtube.com/watch?v=o66ntIeh3rQ&feature=youtu.be or through https://digittools.net/didi-3. DIDI can be downloaded from http://www.digittools.net and NetLogo can be downloaded from https://ccl.northwestern.edu/netlogo/download.shtml**.** All downloads are free.

## Declaration of Competing Interest

The Authors confirm that there are no conflicts of interest.
